# Trends in Health Equity in the United States by Race/Ethnicity, Sex, and Income, 1993-2017

**DOI:** 10.1001/jamanetworkopen.2019.6386

**Published:** 2019-06-28

**Authors:** Frederick J. Zimmerman, Nathaniel W. Anderson

**Affiliations:** 1 Jonathan and Karin Fielding School of Public Health, Department of Health Policy & Management, University of California, Los Angeles

## Abstract

**Importance:**

Health equity is an often-cited goal of public health, included among the 4 overarching goals of the Department of Health and Human Services’ Healthy People 2020. Yet it is difficult to find summary assessments of national progress toward this goal.

**Objectives:**

To identify variation in several measures of health equity from 1993 to 2017 in the United States and to test whether there are significant time trends.

**Design, Setting, and Participants:**

Survey study using 25 years of data, from January 1, 1993, to December 31, 2017, from the Centers for Disease Control and Prevention’s Behavioral Risk Factor Surveillance System to assess trends in health equity and health justice by race/ethnicity, sex, and income in 3 categories by year.

**Main Outcomes and Measures:**

Health equity was assessed separately for each of 2 health constructs: healthy days—the average of physical and mental healthy days over the previous 30 days—and general health in 5 categories, rescaled to approximate a continuous variable. For each health construct, average health was calculated along with 4 measures of health equity: disparities across 3 income groups; black-white disparities; health justice—a measure of the correlation of health outcomes with income, race/ethnicity and sex; and a summary health equity metric.

**Results:**

Among the 5 456 006 respondents, the mean (SD) age was 44.5 (12.7) years; 3 178 688 (58.3%) were female; 4 163 945 (76.3%) were non-Latinx white; 474 855 (8.7%) were non-Latinx black; 419 542 (7.7%) were Latinx; and 397 664 (7.3%) were of other race/ethnicity. The final sample included 5 456 006 respondents for self-reported health and 5 349 527 respondents for healthy days. During the 25-year period, the black-white gap showed significant improvement (year coefficient: healthy days, 0.021; 97.5% CI, 0.012 to 0.029; *P* < .001; self-reported health, 0.030; 97.5% CI, 0.025 to 0.035; *P* < .001). The health equity metric for self-reported health showed no significant trend. For healthy days, the Health Equity Metric declined over time (year coefficient: healthy days, −0.025; 97.5% CI, −0.033 to −0.017; *P* < .001). Health justice declined over time (year coefficient: healthy days, −0.045; 97.5% CI, −0.053 to −0.038; *P* < .001; self-reported health, −0.035; 97.5% CI, −0.046 to−0.023; *P* < .001), and income disparities worsened (year coefficient: healthy days, −0.060; 97.5% CI, −0.076 to −0.044; *P* < .001; self-reported health, −0.029; 97.5% CI, −0.046 to −0.012; *P* < .001).

**Conclusions and Relevance:**

Results of this analysis suggest that there has been a clear lack of progress on health equity during the past 25 years in the United States. Achieving widely shared goals of improving health equity will require greater effort from public health policy makers, along with their partners in medicine and the sectors that contribute to the social determinants of health.

## Key Points

**Question:**

Has health equity improved or worsened during the past 25 years in the United States among working-aged adults?

**Findings:**

Using data from more than 5.4 million respondents to the Centers for Disease Control and Prevention’s Behavioral Risk Factor Surveillance System, this study found that from 1993 to 2017, the black-white gap showed significant improvement. However, measures of health equity and health justice declined over time, and income disparities worsened.

**Meaning:**

Meaningful progress on health equity in the United States will require greater effort, new approaches, or both.

## Teaser

This survey study uses data from over 5.4 million US working-aged adults to assess trends in health equity and health justice by sex, race/ethnicity, and income across the past 25 years.

## Introduction

Health equity is a frequently cited goal of public policy and public health, included among the 4 overarching goals of the Department of Health and Human Services’ Healthy People 2020: National Health Promotion and Disease Prevention and placed at the core of the Robert Wood Johnson Foundation’s Culture of Health Framework. Yet it is difficult to find summary assessments of the extent to which national or state-level progress toward this goal is being made. Without such summary measures, it is difficult to track performance toward health-equity goals.

Although many studies have explored racial/ethnic as well as income disparities in health, no clear picture of the trend of health equity has emerged. One reason is a heavy emphasis on mortality or on specific diseases. However, focusing on any individual mortality or morbidity measure may be an imperfect approximation of general health in the aggregate. Although mortality is correlated with poor health, these are distinct concepts and not perfectly correlated.^1,2,3^ And although a narrow disease focus may help clarify particular problem areas, it may also obscure larger patterns that may be indicative of systemic forces.^4^

By working through the social determinants of health, policy makers in education, criminal justice, the built environment, and other fields influence overall health and not just specific diseases or health behaviors. More walkable neighborhoods, for example, may reduce obesity and cancer risk and may improve both cardiovascular and mental health.^5^ Those who make policy that affects the social determinants of health may accordingly want to know to what extent their efforts are working beyond the traditional measures of their fields, that is, whether overall health and quality of life have become more equal or less equal. This larger question is critical for 2 reasons. First, given limited resources of time, money, and human cognitive capacity, reductions in disparities for one disease may come at the cost of less attention to other areas, so that the total outcomes for overall health, even of equity-enhancing interventions (in particular, diseases), may be less than the outcomes for the targeted disease. As one example, if healthy-eating interventions targeted to one racial/ethnic group come at a cost in psychological well-being for that group,^6,7,8^ there may on the whole be no net improvement in overall health disparities. Second, to the extent that social conditions are fundamental causes of health inequalities,^9^ medical or behavioral health interventions will be inadequate to produce equity in overall health. Improvements in particular diseases over time may simply reflect technological advances, leaving policy makers with the impression that health equity is improving when in fact the underlying inequitable social conditions remain unchanged. In seeking to meet health equity goals, it is essential to track whether these goals are being met overall, not just in narrow disease groups.

The concept of health equity has multiple meanings. Several distinct conceptualizations of health equity exist in the literature: health disparities track differences in health outcomes among 2 or more groups; health inequality tracks the overall variation in health across individuals without regard to social group^10^; a distinct concept—also called *health inequality* in the literature^11^—tracks the extent to which health outcomes are correlated with social attributes, typically economic status. Because this term has referred to 2 very different concepts in the literature, the second concept, the correlation of health with social attributes, is called *health justice* herein. Although this usage is consistent with the existing literature,^12,13^ it is less nuanced, and for this reason makes no claim to supplant any of the other existing concepts of health justice.

A summary definition of health equity attempts to unite these distinct concerns into a single construct^14^: health equity means that everyone has a fair and just opportunity to be as healthy as possible. The definition also clearly emphasizes health as distinct from life expectancy or access to health services and is consistent with the way policy makers understand health equity.^15^ The purpose of the present work is to identify trends in health equity over time. A clear sense of the evolution of health equity may clarify whether and to what extent progress is made, or whether greater efforts are required.

## Methods

### Study Data

This study followed the Strengthening the Reporting of Observational Studies in Epidemiology (STROBE) reporting guideline.^16^ We used 25 years of data from the Centers for Disease Control and Prevention’s (CDC’s) Behavioral Risk Factor Surveillance System (BRFSS), from January 1, 1993, to December 31, 2017. These waves are all of those for which data on the main outcomes were available. The study was deemed exempt from review by the University of California, Los Angeles institutional review board, which waived the need for informed consent owing to use of deidentified data.

The sample included all respondents aged 18 to 64 years to reflect the health experience of working-aged US adults. Although data for older US adults are available, the focus in this analysis is on the working-aged population because of the different determinants of health among persons of working age and those 65 years and older. All 50 states and the District of Columbia were included. Four states did not participate in BRFSS for 1 year of the study period: Wyoming (1993), Rhode Island (1994), the District of Columbia (1995), and Hawaii (2004).

### Main Outcomes and Measures

Two self-reported concepts of health were used: general health on a 5-point scale (excellent; very good; good; fair; and poor) and the mean number of healthy days for physical and mental health during the past 30 days. These measures have been shown to capture overall health-related quality of life well,^17^ and they have good psychometric properties, including high concurrent validity with other measures,^18^ high predictive validity,^18,19^ validity in a variety of subpopulations,^20,21,22,23,24^ and good test-retest reliability.^18,25^ For these reasons, these measures are recommended by the CDC for assessment of population health.^18^

For the purposes of this analysis, self-reported general health was scaled using a procedure previously recommended,^23^ (eAppendix 1 in the Supplement).

The BRFSS includes demographic and economic questions that are used as the basis of the health equity measures. Respondents reported household income in 7 or 8 categories depending on the year. The cutoffs for these income categories are periodically adjusted by the CDC to reflect inflation. Responses were placed into 3 categories to achieve relative stability of percentages in the 3 categories across the years. Missing responses were placed in the middle category. The sample proportions in the highest income group (over the 25 years) had a mean (SD) of 26% (4.7%) (range, 16.0% in 2000 to 32.0% in 2017). Race/ethnicity was coded into 4 mutually exclusive and collectively exhaustive categories: white non-Latinx; black non-Latinx; Latinx; and other. Those with missing race/ethnicity data were categorized as other.

For each of the 2 concepts of health—self-reported general health and healthy days—and for the nation in each year, we calculated mean health and the following 4 measures of health equity:

- Black-white disparities were calculated as the mean of the health outcome variable for white non-Latinx individuals minus the average value for black non-Latinx individuals.
- Income disparities were calculated using the mean difference in outcomes between the top, middle, and bottom income categories. This measure has been shown to be similar to other measures comparing multiple groups.^26^
- Health justice reflects the extent to which health outcomes are correlated with identifiable social attributes of sex, income, and race/ethnicity. It is calculated in 2 steps. First, the health outcome is regressed on age alone. Second, the health outcome is regressed on age, sex, income, and race/ethnicity. The difference in 2 *R*^2^ values from these 2 regressions is then subtracted from 1 to produce a measure that ranges from 0 to 1 and for which higher values mean that less of the variation in health outcomes is explained by sex, income, and race/ethnicity, thereby implying greater health justice.
- The health equity metric, for which further technical details are available on request, integrates health inequality, health disparities, and mean health into a single, summary measure. It can be understood as the mean weighted departure of individual health from the best achievable health for each year, in which the weighting scheme is such that larger departures from the best achievable health are weighted more heavily than smaller departures. The best achievable health is defined as the median of the most privileged identifiable group, here white men in the top income category. The formula for the health equity metric is as follows:

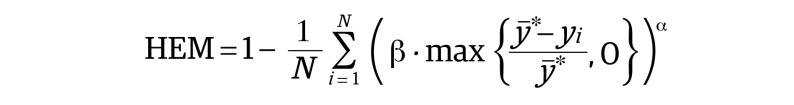
in which HEM is the health equality metric, *N* is the total number of individuals in the sample, *y_i_* is an individual’s health, y̅* is the median health in the most-privileged group, and β and α are parameters, with α greater than 2 and β greater than 0. This measure can be considered an average distastefulness measure, in which the distastefulness of poor health increases more than proportionately with departures from optimal health. This measure is important for integrating into an overall measure those health disparities that cannot be captured explicitly in the data, including for particular social groups (eg, Muslim individuals, lesbian, gay bisexual, trans, and queer/questioning [LGBTQ] individuals, undocumented immigrants), for those for whom marginalization may be the result of intersectionality, and those for whom sample sizes are not large enough to reliably estimate health outcomes (eg, Native American individuals). Although the experiences of any one of these groups cannot be disentangled from the overall measure of health equity, this measure is distinct from the summary measures typically used in that it does not claim to be representative of heterogeneous categorizations.

### Statistical Analysis

Each of the mean health and health equity measures was calculated for the nation as a whole and for each of the state-year combinations for which data were available. State-level measures are not the focus of this analysis, but they were calculated here to help contextualize the national results. Scatterplots of state-year outcomes for each of the health equity measures described herein are presented.

Sampling weights were used,^27^ and the estimates were age-adjusted using the following age strata: 18 to 29, 30 to 39, 40 to 49, 50 to 56, and 57 to 64 years. These strata were chosen to be large enough to ensure adequate sample size within age strata (and racial/ethnic and income groups) and small enough for within-strata homogeneity of outcomes.^18^ The standard population was the US population in the halfway point of the period, 2004, using the 1-year American Community Survey Public Use Microsample. Black-white disparities were calculated only when there were 3 or more black respondents in each age category in the state-year.

In 2011, BRFSS underwent a redesign that included the adoption of raking as a statistical weighting method and the addition of cellular-telephone households. In general, these changes increased prevalence estimates of poor health, although the outcomes associated with these changes vary from state to state.^28^ To adjust for this change, we subtracted the 2010 minus 2011 differences on each measure from the estimates before 2011. This procedure introduces a bias toward the null in tests of trends in the measures over time.

To test for time trends, each of the measures was regressed on year as a continuous variable at the national level. Because the purpose of this work is to identify trends in health equity and not to isolate causal factors, the regressions are sparse: no controls were added for potential reasons for trends in health equity, such as economic, political, or population changes. To control for the different proportions of the population in the highest and lowest income categories, these variables were added as potential confounders to the regressions of income disparities on year. Because there are 2 health outcomes, with no a priori reason to expect distinct trends, a Bonferroni correction was used. Statistical tests are accordingly 2-sided tests with an α of .025. All analyses were conducted in Stata statistical software version 15 (StataCorp LLC).

## Results

Among the 5 456 006 respondents, the mean (SD) age was 44.5 (12.7) years; 3 178 688 (58.3%) were female; 4 163 945 (76.3%) were non-Latinx white; 474 855 (8.7%) were non-Latinx black; 419 542 (7.7%) were Latinx; and 397 664 (7.3%) were of other race/ethnicity. The healthy days questions were omitted from the survey for 29 states in 2002. The final sample included 5 456 006 respondents for self-reported health and 5 349 527 respondents for healthy days.

eAppendix 2 in the Supplement shows pairwise correlations of the various measures of health equity across all of the state-year combinations. Most of the correlations between measures are moderate, suggesting that the concepts of health equity are empirically distinct from one another (eAppendix 2 in the Supplement).

**Figure 1.**
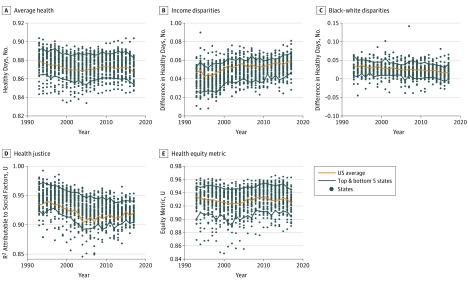
Health-Equity Trends by Self-reported Health

**Figure 2.**
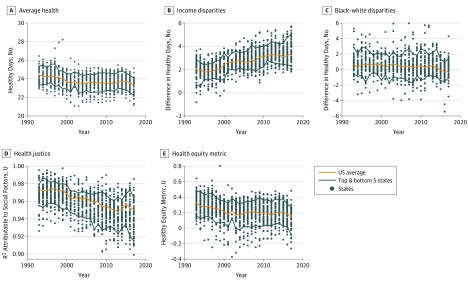
Health-Equity Trends by Healthy Days

Figure 1 shows the trends for average health and health equity measures for the scaled self-reported general health measures. Figure 2 shows these trends for the healthy days measure. In each figure, the state-year scatterplots are shown in the background for context. The difference between the mean number of healthy days between the top and bottom income categories had an unweighted mean (SD) across states of 2.0 (0.9) days in 1993, increasing to 3.7 (1.0) days in 2017. The difference between the mean number of healthy days between white individuals and black individuals had a mean (SD) of 0.9 (1.5) days in 1993, decreasing to 0.0 (1.2) days in 2017. The health justice measure had a mean (SD) of 0.97 (0.01) *R*^2^ units in 1993, decreasing to 0.94 (0.02) units in 2017. The Health Equity Metric had a mean (SD) of 0.32 (0.13) units in 1993, increasing to 0.13 (0.15) units in 2017. The difference of the mean scaled value of self-reported health between the top and bottom income categories had an unweighted mean (SD) across states of 0.042 (0.011) units in 1993, increasing to 0.057 (0.009) units in 2017. The difference of the mean scaled value of self-reported health between white individuals and black individuals was a mean (SD) of 0.030 (0.020) units in 1993, decreasing to 0.019 (0.017) days in 2017. The health justice measure had a mean (SD) of 0.94 (0.02) *R*^2^ units in 1993, decreasing to 0.92 (0.01) units in 2017. The health equity metric had a mean (SD) of 0.932 (0.019) units in 1993, increasing to 0.926 (0.016) units in 2017.

The Table reports the national-level regressions of each measure on year. To facilitate comparisons across measure in this table only, each measure was rescaled by dividing by the interquartile range across states in 1993. The black-white gap and income disparities were reverse-coded so that higher values imply greater health equity for all measures.

**Table.** National Estimates of Change in Health-Equity Constructs From 1993 to 2017^a^

Equity Measure	Years, No.	Year Coefficient (97.5% CI)	*P* Value
**Healthy Days**			
Average health	25	−0.023 (−0.032 to −0.015)	<.001
Black-white health gap	25	0.021 (0.012 to 0.029)	<.001
Income disparities	25	−0.060 (−0.076 to −0.044)	<.001
Health justice	25	−0.045 (−0.053 to −0.038)	<.001
Health equity metric	25	−0.025 (−0.033 to −0.017)	.001
**Self-Reported Health**			
Average health	25	−0.017 (−0.029 to −0.006)	.005
Black-white health gap	25	0.030 (0.025 to 0.035)	<.001
Income disparities	25	−0.029 (−0.046 to −0.012)	.002
Health justice	25	−0.035 (−0.046 to −0.023)	<.001
Health equity metric	25	0.001 (−0.007 to 0.009)	.84

^a^ Each row represents a separate regression, with the outcome listed in the left column, as scaled by fraction of the interquartile range in 1993 across states. The black-white gap and income disparities were reverse coded: for all outcomes, higher values imply greater health equity. In each regression, year was the only covariate, except in the regression of income disparities, which included controls for the proportion of the population in the highest and lowest income categories.

For both self-reported health and healthy days, mean health has been trending downward over time (year coefficient: healthy days, −0.023 [97.5% CI, −0.032 to −0.015]; *P* < .001; self-reported health, −0.017 [97.5% CI, −0.029 to −0.006]; *P* = .005).

Income disparities increased across the study period, and the change over time was greater compared with within-year variation across states. The time trend for income disparities (reverse-coded) for both health concepts was significantly negative (ie, toward less equity), with a year coefficient for healthy days of −0.060 (97.5% CI, −0.076 to −0.044; *P* < .001); and a year coefficient for self-reported health of −0.029 (97.5% CI, −0.046 to −0.012; *P* < .001).

By contrast, black-white disparities were small and trending slightly downward over time for both outcomes. Over the 25-year period, the black-white gap showed significant improvement (year coefficient: healthy days, 0.021 [97.5% CI, 0.012 to 0.029]; *P* < .001; self-reported health, 0.030 [97.5% CI, 0.025 to 0.035]; *P* < .001).

Health justice, a measure of the independence of health outcomes from race, sex, and income, displayed similar but somewhat different patterns for healthy days and self-reported health. Health justice decreased generally throughout the period for healthy days, with a modest improvement from 2012 to 2015, gains that were erased by 2017. Overall, though, the trend was downward, with the year coefficient for healthy days of −0.045 (97.5% CI, −0.053 to−0.038; *P* < .001) and the year coefficient for self-reported health of −0.035 (97.5% CI, −0.046 to −0.023; *P* < .001).

The health equity metric for self-reported health showed no significant trend. For healthy days, the health equity metric declined over time (year coefficient: −0.025 [97.5% CI, −0.033 to −0.017]; *P* < .001).

## Discussion

The results of this study show a worrisome lack of progress on health equity during the past 25 years in the United States. Although there are some differences across conceptualizations of health equity, and small differences across the 2 concepts of overall health, the overall pattern is one of stagnation mixed with unambiguous decline.

Much previous work has focused on mortality. The results here, although focusing on general health rather than mortality, are consistent with this previous work. One study found a narrowing in national black-white disparities in all-cause mortality from 1990 to 2005.^4^ From 2001 to 2014, income disparities in life expectancy increased,^29^ and an analysis of disparities in life expectancies across counties found that they have been increasing since 1980 and were correlated with county-level racial/ethnic proportions and income.^30^ Such results are in line with the results for increasing income disparities in general health as reported herein. Also similar is an analysis of education-related disparities in mortality across states, which found wide differences across states, coupled with an overall pattern of minimal progress.^31^

Looking at trends in several concepts of health equity using broad concepts of health reveals an overall pattern that provides important context to studies focusing on particular diseases and conditions. To cite a handful of the literature, studies have found little change in black-white differences in obesity since 1980^32^ and in preventable hospitalizations^33^ (2001-2009); modest declines in tuberculosis from 1993 to 2010^34^; and a mixed pattern of both increases and decreases in racial/ethnic disparities in diabetes from 2006 to 2010.^35^

### Limitations

This study has limitations. A report by the National Research Council noted that data necessary to track health disparities are limited.^36^ Even with its large sample size, the present analysis is not able to specifically identify additional measures important to the understanding of health equity, such as those involving religious, sexual, or immigration-status minorities or those dimensions that arise from intersectionality.

Estimations of income disparities were hobbled by a lack of continuous income data, so that the relative definition of income categories changes across time, although not systematically with time. These are common problems in studies of disparities and equity over time.^37^ Controls for the relative sizes of income categories in the regression of income disparities on year help to mitigate this problem. eAppendix 3 in the Supplement provides additional detail.

In these data, mean health and the health equity metric are correlated, because a large fraction of the sample is clustered around maximum self-reportable health levels: a value of “excellent” in self-reported general health and a value of 30 on the healthy days measures. A health outcome with a more normal distribution would show greater separation between these measures.

Notwithstanding these limitations, the relative consistency of the study’s overall finding of limited progress on health equity suggests some important implications. The first is that the country as a whole must work harder to promote health equity if the often-stated goals of improving health equity are to be met. Second, an area of particular focus in the future should be understanding which collection of policies would produce the greatest improvement in health equity. The analysis here hints that increasing income disparities may be associated with stagnant health equity. If this is in fact the case, policies that reduce the prevalence and penalties associated with poverty would be a clear starting point to improving health equity.

## Conclusions

Improving health equity often figures as an important goal for communities, thought leaders, and policy makers in public health. Yet, this analysis suggests that across the past 25 years, the promise of improving health equity has not been met. Greater or different efforts than those tried in the past will have to be mustered if health equity is to improve. Performance tracking of health equity may help to keep policy makers accountable to making the necessary changes.

## Notes

Supplement. **eAppendix 1.** Scaling of Self-Reported General Health **eReferences.** **eAppendix 2.** Correlation Matrices of the Measures for Scaled Self-Rated Health (A) and Measures for Healthy Days (B) **eAppendix 3.** Change in the Sizes of Income Categories
