# Incorrect Wording in Results

**DOI:** 10.1001/jamanetworkopen.2019.9357

**Published:** 2019-07-24

In the Original Investigation titled “Trends in Health Equity in the United States by Race/Ethnicity, Sex, and Income, 1993-2017”^1^ published June 28, 2019, the changes in mean (SD) scores for the health equity metric between 1993 and 2017 for both Figure 1 and Figure 2 were incorrectly described as “increasing” on initial publication. In the third paragraph of Results, the sixth sentence should have read, “The Health Equity Metric had a mean (SD) of 0.32 (0.13) units in 1993, decreasing to 0.13 (0.15) units in 2017,” and the 10th sentence should have read, “The health equity metric had a mean (SD) of 0.932 (0.019) units in 1993, decreasing to 0.926 (0.016) units in 2017.” This article has been corrected.^1^
