# Supplementary material for: Trends in Health Equity in the United States by Race/Ethnicity, Sex, and Income, 1993-2017
Source: JAMA Netw Open. 2019 Jun 28;2(6):e196386. doi: 10.1001/jamanetworkopen.2019.6386 (PMC6604079; doi:10.1001/jamanetworkopen.2019.6386)
Supplement: Supplement. — eAppendix 1. Scaling of Self-Reported General Health eReferences. eAppendix 2. Correlation Matrices of the Measures for Scaled Self-Rated Health (A) and Measures for Healthy Days (B) eAppendix 3. Change in the Sizes of Income Categories [file jamanetwopen-2-e196386-s001.pdf]

Supplementary Online Content

Zimmerman FJ, Anderson NW. Trends in health equity in the United States by race/ethnicity, sex, and income, 1993-2017. *JAMA Netw Open*. 2019;2(6):e196386. doi:10.1001/jamanetworkopen.2019.6386

eAppendix 1. Scaling of Self-Reported General Health

eReferences.

eAppendix 2. Correlation Matrices of the Measures for Scaled Self-Rated Health (A) and Measures for Healthy Days (B)

eAppendix 3. Change in the Sizes of Income Categories

This supplementary material has been provided by the authors to give readers additional information about their work.

© 2019 Zimmerman FJ et al. *JAMA Network Open.*

eAppendix 1. Scaling of Self-Reported General Health

It is possible to assign appropriate weights to the self-reported general health responses to approximate a continuous variable, for each equal-increment changes have similar meanings to health.[1-3] (Doing so is in general preferable to the alternative approach of dichotomizing the underlying variable, which loses important information and is accordingly statistically inefficient.) This study uses the preferring weighting scheme of Doorslaer and Jones (2003), in which results of an analysis of health inequality from this weighting scheme are shown to be nearly identical to the results from an analysis using a full health utilities instrument. The weighting scheme is shown below.

**Self-reported general health response category**	**Assigned Weight**
Poor	0.401
Fair	0.707
Good	0.841
Very Good	0.931
Excellent	0.983

eReferences.

1. Wagstaff A, Van Doorslaer E. Measuring inequalities in health in the presence of multiple-category morbidity indicators. Health economics. 1994;3(4):281-291.
2. DeSalvo KB, Fisher WP, Tran K, Bloser N, Merrill W, Peabody J. Assessing Measurement Properties of Two Single-item General Health Measures. Quality of Life Research. 2006;15(2):191-201.
3. Doorslaer Ev, Jones AM. Inequalities in self-reported health: validation of a new approach to measurement. Journal of Health Economics. 2003;22(1):61-87.

© 2019 Zimmerman FJ et al. *JAMA Network Open.*

eAppendix 2. Correlation Matrices of the Measures for Scaled Self-Rated Health (A) and Measures for Healthy Days (B)

A. Measures for Scaled Self-Rated Health				
Measure	Average Health	Income Disparities	Black-White Disparities	Health Justice
Income Disparities	-0.62			
Black-White Disparities	0.22	-0.18		
Health Justice	0.20	-0.64	-0.09	
HEM	0.90	-0.58	0.23	0.11

B. Measures for Healthy Days				
Measure	Average Health	Income Disparities	Black-White Disparities	Health Justice
Income Disparities	-0.60			
Black-White Disparities	0.13	-0.09		
Health Justice	0.38	-0.70	<0.01	
HEM	0.95	-0.58	0.18	0.37

© 2019 Zimmerman FJ et al. *JAMA Network Open.*

eAppendix 3. Change in the Sizes of Income Categories

The income categories vary somewhat over time, with the highest income category encompassing the smallest proportion of the population (15.9%) in 2000, and the largest proportion (32.1%) in 2017. There is a sharp change from 1999 (29.0%) to 2000 (16%), which offers an opportunity to gauge the impact of the size of the top income category. Between these years, the change in the disparity is about 3 times the average absolute change between other adjacent years for the healthy days outcome, and about equal to the average absolute change for the scaled general health outcome.

© 2019 Zimmerman FJ et al. *JAMA Network Open.*
